# Analytical Models for Pose Estimate Variance of Planar Fiducial Markers for Mobile Robot Localisation

**DOI:** 10.3390/s23125746

**Published:** 2023-06-20

**Authors:** Roman Adámek, Martin Brablc, Patrik Vávra, Barnabás Dobossy, Martin Formánek, Filip Radil

**Affiliations:** 1 Faculty of Mechanical Engineering, Brno University of Technology, Technická 2896/2, 616 69 Brno, Czech Republic; 2 Independent Researcher, 74 401 Frenštát pod Radhoštěm, Czech Republic

**Keywords:** planar fiducial marker, robot localisation, Kalman filter, observation noise

## Abstract

Planar fiducial markers are commonly used to estimate a pose of a camera relative to the marker. This information can be combined with other sensor data to provide a global or local position estimate of the system in the environment using a state estimator such as the Kalman filter. To achieve accurate estimates, the observation noise covariance matrix must be properly configured to reflect the sensor output’s characteristics. However, the observation noise of the pose obtained from planar fiducial markers varies across the measurement range and this fact needs to be taken into account during the sensor fusion to provide a reliable estimate. In this work, we present experimental measurements of the fiducial markers in real and simulation scenarios for 2D pose estimation. Based on these measurements, we propose analytical functions that approximate the variances of pose estimates. We demonstrate the effectiveness of our approach in a 2D robot localisation experiment, where we present a method for estimating covariance model parameters based on user measurements and a technique for fusing pose estimates from multiple markers.

## 1. Introduction

The field of mobile robotics is experiencing a period of dynamic development, as can be seen from the ongoing research and the growing number of commercial applications [1,2]. One of the cornerstones of this field is the problem of robot localisation, i.e., determination of the robot’s pose (position and orientation) with respect to its surrounding environment. Based on the robot’s operating environment, we distinguish between indoor and outdoor robot localisation.

The problem of long-term outdoor localisation was significantly simplified by the ability to use the global navigation satellite system (GNSS). However, long-term indoor localisation remains a challenging task [3]. A common approach to indoor robot localisation is based on landmarks. Landmarks are distinct features of the environment that can be of natural or artificial origin. Natural landmarks, such as corners, columns and doors, are built-in features of the indoor environment, which is their main advantage and many localisation and mapping approaches rely on them [4]. However, they come at a cost of being less robust; according to Jang et al. [4] they are not unique, susceptible to viewing angle, background clutter and also environment variations, such as lighting conditions. On the other hand, artificial landmarks require modifications in the interior environment; they are designed to be easily detected and extracted from the environment [5].

Artificial landmarks can be either active or passive. Active landmarks emit information into the environment and can be represented by devices already present in the environment, such as WLAN [6] or specially designed devices (ultra-wideband radio [7] or optical beacons [8]). These devices are rather complex and expensive, and require a power source. In contrast, passive markers (also known as fiducial markers) are an inexpensive and simple way to obtain a relative positional reference from the sensor, usually the camera, to the marker. This might be useful in situations where we need a relative localisation between the robot and, for example, a charging station. If we already know the global position of the marker in the environment, we can obtain the global position of our robot.

### 1.1. Passive Fiducial Markers

Passive fiducial markers come in different spatial dimensions, shapes and sizes. For mobile robotics, planar quadrilateral markers (e.g., ArUco [9], AprilTag, ARTag) are among the most commonly used variants described in [10,11]. These markers are monochromatic, and the information is encoded in n-by-n internal pixels that are surrounded by a frame designed to help distinguish them from their surroundings and used to estimate the position of the marker relative to the camera. The data encoded in the internal pixels usually contains an identifier and some additional information that is used to check redundancy and recover lost information [5]. Practical applications of these markers are:

- Kinematic calibration for industrial robots;
- Visual servoing of industrial robots;
- Robot navigation tasks;
- Localisation and mapping tasks;
- Human–machine interaction.

Extracting the information encoded in a marker requires several processing steps. In the first step, the processing algorithm focusses on finding the marker’s frame (also known as an anchor) in the image, which is usually a geometric shape such as a point, bar, ellipse, triangle or square. For quadrilateral systems, the anchors consist of four lines. The next step is to extract the polygons from the inner pixels, which are then merged into a larger code pattern. The extracted pattern is then validated against a library of possible code schemes. In the last step, the six-degrees-of-freedom (DoF) pose of the marker in the camera reference frame is estimated by computing the homographic transform [12,13]. Like other solutions for mobile robot localisation, planar fiducial markers have their own shortcomings:

- *Pose ambiguity* [14,15]: during frontal observation of the marker the pose cannot be determined uniquely, i.e., the valid and the flipped invalid orientation are indistinguishable [14];
- *Degradation of accuracy with distance* [16];
- *Occlusion* [17,18]: this arises when a real object appears between the camera and a fiducial marker, making part of the marker invisible to the camera—it causes problems with marker detection
- *Marker size*: according to Szentandrási et al. [17] (p. 1) “the markers must be large enough to provide sufficient amount of information and at the same time they must be small enough to fit into the camera’s field of view”;
- *Optical system limitations* [5]: physical properties of the image acquisition system, e.g., resolution.

The two former issues, namely degradation of orientation accuracy in frontal observation and pose ambiguity, arise as the result of the estimation principle used to determine the pose of the marker. These problems are widely known, but the methods currently available do not provide a real solution; they merely mitigate their effects [14]. The presence of these issues is particularly relevant in mobile robotics, as incorrect pose information from a marker can corrupt localisation and present a risk to the integrity of the robot itself [14].

### 1.2. Extended Kalman Filter

Much like the above-mentioned issues related to marker pose estimation, measurements from other sensors also suffer from inaccuracies and contain a non-negligible amount of uncertainty. In order to deal with measurement inaccuracies and incorporate the related uncertainty, state estimator algorithms are used. One of the most commonly used is the Kalman filter (KF) [19] and its extension for nonlinear systems, the Extended Kalman Filter (EKF) first described in [20]. The advantage of the EKF is that it allows the combination (also the correction) of measurements from different sensors (inertial measurement unit (IMU), GNSS, odometry), thus increasing the accuracy of the estimate of the monitored quantities (in the case of a mobile robot, it is usually the robot’s pose, speeds and accelerations). In order for the EKF to work correctly, we need to have a model of the observed system describing the state $g(u_{k},x_{k-1}$), measurement prediction $h(x_{k})$, and the related uncertainty of process noise and observation noise given by covariance matrices $Q_{k}$ and $R_{k}$, respectively. Here $u_{k}$ denotes control data, while $x_{k}$ and $x_{k-1}$ mark state vector at time *k* and k−1 respectively.

**Figure 1:**
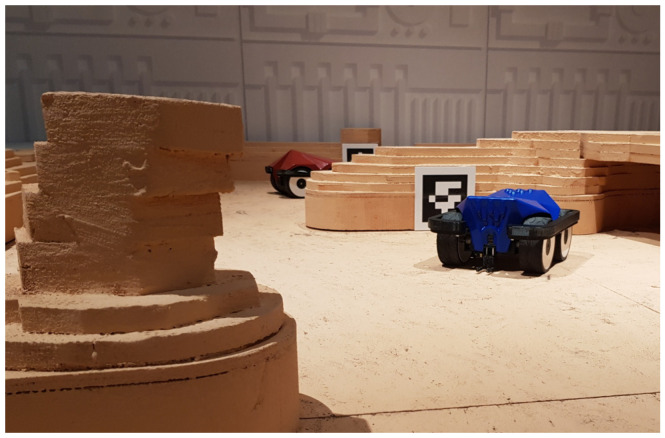
Depiction of mobile robots in a described environment.

In practical applications, the determination of covariance matrices $Q_{k}$ and $R_{k}$ is not straightforward and is often set using the trial and error approach [21]. In this work, we will present a method that addresses the issue of setting the variance of the measurement input from the planar fiducial markers and how to use it in a practical scenario of localisation of mobile robots operating in a 2D environment with known global positions of fiducial markers as shown in Figure 1.

## 2. Related Works

Planar fiducial markers were first mentioned in relation to pose estimation at the end of the last millennium. In their article, Tung et al. [22] used simple fiducial marks to locate the origin of parts’ local coordinate frame in an autonomous assembly task. In 1998 Rekimoto [23] presented an approach for fiducial marker processing, which combined pattern recognition and pose estimation; soon other applications followed, e.g., optical tracking, Sticker et al. [24], and augmented reality [25]. The article by Kato et al. [25] also introduced the first fiducial marker system called ARToolKit. Soon other marker systems started to emerge (e.g., ARTag [26], AprilTag [27] and STag [28], ArUco [9,29]) that have been designed to mitigate some of the shortcomings of the ARToolkit.

Of the aforementioned shortcomings, pose ambiguity during frontal observation (i.e., small viewing angle) is one of the key issues [14]; it is caused by the mathematical apparatus used to process the markers. Perspective projection is used to estimate the shape distortion of a marker in an image. As a result, the larger the angle of view, the more accurate the pose estimation. In contrast, from near-frontal observation, the pose estimation produces high uncertainty. Tanaka et al. [14] (p. 2) describe this phenomenon as “close to an orthogonal projection, sometimes we cannot distinguish the valid orientation and the flipped invalid orientation because they seem almost the same in the image”. More details on this topic are available in [14].

Several articles have addressed the question of the pose ambiguity. In [5], Posescu et al. tested the accuracy of pose estimation from ArUco markers and noted that the uncertainty is the largest for frontal observation of the marker. The previous findings are also supported by Lopez-Cerón et al. [30]. The authors performed experiments in which they examined the effects of different observation distances and angles in relation to the AprilTag marker. Similar results to previous papers were presented in [31,32] but, in these cases, the issue was observed on other types of markers, the ARToolKit marker and the Metaio marker, respectively, and experiments were carried out with a finer angular and distance resolution. In [32] it was shown that higher standard deviation values occur not only for frontal observations but also for observations under a large angle of view. In [16], the authors proved that not only AprilTag and ArUco, but also other marker systems such as ARTag and STag, are to a greater or lesser extent susceptible to these shortcomings.

To address these issues, different approaches were proposed. Some of the articles proposed new marker systems; for example, Tanaka et al. [33] proposed markers based on Moire patterns. Other authors proposed larger markers in combination with alternative patterns such as random dots proposed by Uchiyama et al. [34] or the uniform marker field proposed by Szentandrási et al. [17]. Another group of articles recommends the use of 3D markers [5,13,35]; however, the usage of these markers might be inconvenient, e.g., larger spatial requirements or a geometric model has to be very accurate [33].

Finally, there are articles that recommend the use of fusion algorithms (e.g., extended Kalman filter). These algorithms either combine pose estimates from fiducial markers with readings from a complementary sensor or pose estimates derived from two or more markers. An interesting implementation of the latter solution is presented in [30], where the authors propose an algorithm that determines the final position of the robot based on independent estimates of angular and coordinate values from multiple markers. In articles using the EKF, it is common that data from the motion sensor are typically included in the prediction step, while the pose estimate from the markers is used in the correction step. However, the type of sensors used in the EKF prediction model and the type of markers may differ from article to article; for example, Zeng et al. [36] used encoders, in [10,11,18] IMU is used, while in [37] Chavez et al. used the combination of IMU and Doppler velocity log (DVL). The correct setting of the observation noise covariance matrix is also essential for the proper function of the Kalman filter. Chavez et al. and Zheng et al. used a diagonal covariance matrix. Chavez et al. used as matrix entries the standard deviation of pose estimates from several markers, while Zheng et al. adjusted the entries proportionally to the estimated distance and angle of view of the markers. In [10,11,18], there is little information on how the authors have addressed this issue.

This article builds on the research of Patrik Vávra (co-author of the article), presented in his master thesis [29]. In this paper, we propose analytical functions that model the measurement noise of the pose estimates obtained from the planar markers. The main contribution is that these models reflect the true nature of measurement noise and the outputs of these models can be used to set values in the observation noise covariance matrix $R_{k}$ in EKF which does not need to be set using the trial and error method. The adaptive adjustments of $R_{k}$ mean that EKF can rely more on other sensors when the pose estimate obtained from markers is reliable and vice versa.

## 3. Problem Definition

As briefly described in Section 1, methods presented in this work are related to the problem of global localisation of an autonomous mobile robot in a 2D environment. Mobile robots that were the focus of this research are shown in Figure 1. These robots are four-wheel skid steer robots equipped with a colour camera set to a resolution of 1280 × 720 px, IMU and wheel encoders on each wheel. These robots were moving in a known 2D environment with scattered AruCo markers. The position of these markers relative to the global coordinate system is known and can therefore be used to provide a global positional estimate.

**Figure 2:**
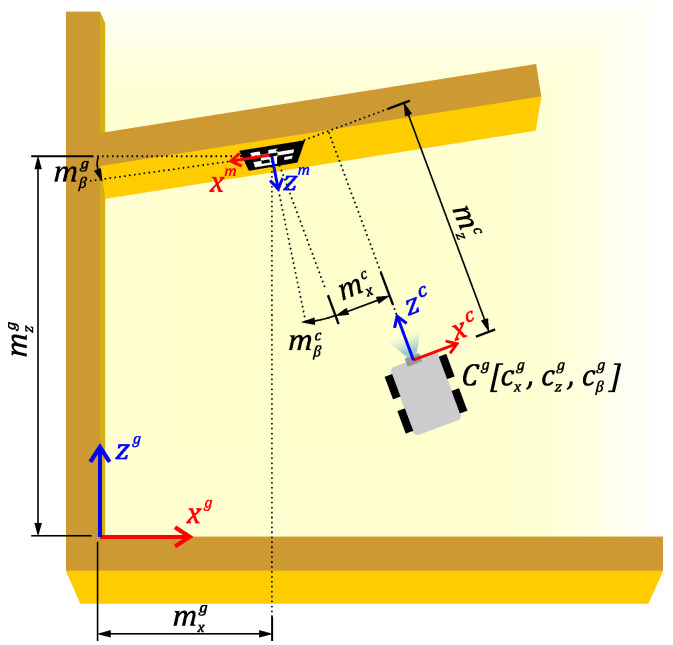
Schematic view of the real application with individual coordinate systems and dimensions. Each coordinate system is marked with an upper index, *g* for the global coordinate system, *m* for the marker coordinate system and *c* for the camera coordinate system.

The schematic view of the environment is shown in Figure 2. From each marker observation, an estimate of the position and orientation of the marker in a 3D space relative to the camera is obtained. However, for localisation in a 2D plane, only the position in the x−z plane and rotation around the *y* axis denoted as β are important. In Figure 2, the known position of the marker, expressed by position $m_{x}^{g}$, $m_{z}^{g}$ and rotation $m_{\beta }^{g}$, is shown in the global coordinate system, as well as the measured position of the marker, denoted as $m_{x}^{c}$, $m_{z}^{c}$ and $m_{\beta }^{c}$, in the camera coordinate system. The goal of this application is to determine the position of the camera $C^{g}$ in the global coordinate system together with its respective covariance matrix that can be used as input in the EKF.

## 4. Experimental Measurements of Pose Estimation Noise

This section describes the conducted experimental measurements in the real world and simulation. The results of these measurements serve to validate the character of measurement noise of the camera pose estimates obtained from the markers in different setups and compare them with results from previous research. Furthermore, the results will serve as a basis for the derivation of variance models presented in Section 5.

### 4.1. Experiment Setup

The goal of the following experiments is to present how the shortcomings of the planar markers, discussed in Section 1, affect the pose estimate obtained from the marker in relation to the mutual position of the marker and the camera. To achieve this, the camera was positioned in a grid relative to the marker and, at each position, the marker was rotated around its vertical axis $y^{m}$.

During the measurements, the ArUco marker with ID 0, resolution 5 × 5 and edge size of 112 mm was mounted on a stepper motor, allowing a precise and repeatable rotation of the marker from $-90^{\circ }$ to $+90^{\circ }$ with the step of $0.45^{\circ }$. A Logitech C922 camera with a resolution set to 1280 × 720 px was used to take 100 photos of the marker at each step of its rotation. In total, 40,100 photos were taken during a single experiment. Symmetrical and diffuse light was used to provide even illumination of the marker to minimise the effect of uneven light and shadows on the results.

**Figure 3:**
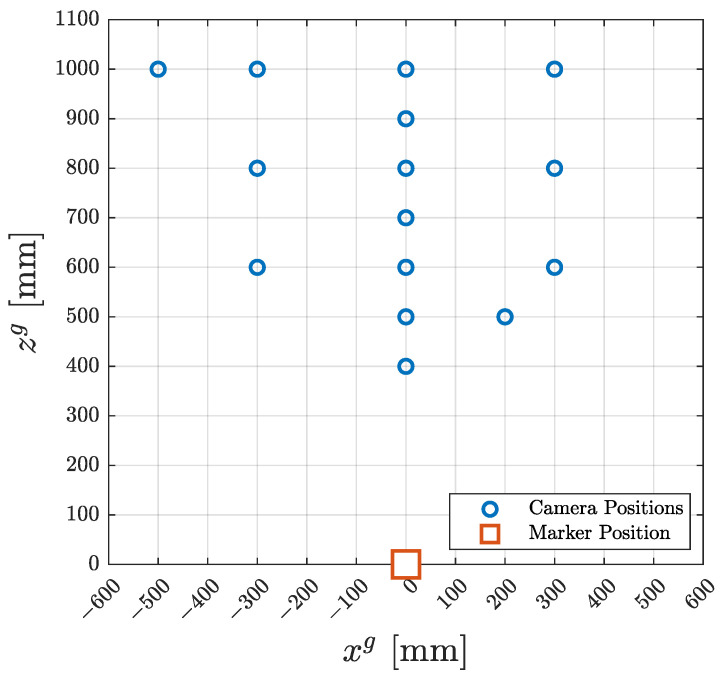
Camera positions during the experimental measurements.

The same experiment was repeated 15 times for different positions of the camera relative to the marker, as shown in Figure 3. In seven experiments, the camera was in line with the marker, the distance between them increasing from 400 mm to 1000 mm. In the rest of the experiments, the camera was placed off-centre from the marker in the direction of the $x^{g}$ axis.

All measurements were processed in Python 3.8.7 using the ArUco library included in OpenCV [38] in version 4.5.1. The camera was calibrated using a chequerboard pattern and functions available in OpenCV. Functions for marker detection and pose estimation were used with default settings; only the corner refinement method was set to the sub-pixel option to improve the pose estimation.

In the post-processing phase, the consecutive positions of the marker corners found in the photo are obtained. The positions of the four corners $u_{i}$ and $v_{i}$, *i* being the index of the corner, are expressed in the coordinate system of the photo with axes *u* and *v*. Based on these positions, the area of the marker in the photo can be calculated using Equation (1) representing Gauss’s area formula. Detected corners are also used to obtain an estimated marker pose in the form of a rotation vector and a translation vector. The rotation vector is subsequently converted to Euler angles, which will be used to represent the rotation of the marker in the rest of this work.

(1) $$S=\frac{1}{2}|u_{1}v_{2}+u_{2}v_{3}+u_{3}v_{4}+u_{4}v_{1}-u_{2}v_{1}-u_{3}v_{2}-u_{4}v_{3}-u_{1}v_{4}|$$

### 4.2. Measurement Results

As mentioned in Section 3, for the case of robot localisation in a 2D space, the required results from marker pose estimation are position in the x−z plane and rotation around the *y* axis. Therefore, only these results will be further presented and analysed.

**Figure 4:**
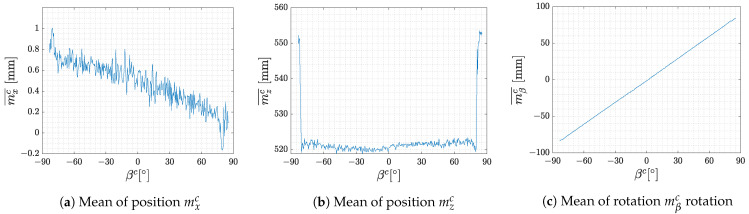
Mean values in individual increments of marker headings.

The following figures present the mean values and variances of an experiment in which the marker was placed 500 mm from the camera in $z_{c}$ and 0 mm in $x_{c}$. All values are displayed in dependence on the angle $\beta^{c}$ set by the stepper motor, which we consider as a ground truth. Figure 4 presents the mean values of the position and rotation of the marker relative to the camera. It can be seen that the marker is detectable approximately in the range of $\pm 80^{\circ }$ but deviations of the estimated position can be observed at the edges of the measurement range.

**Figure 5:**
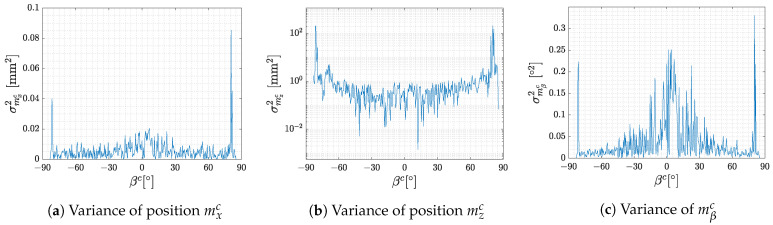
Variance of marker position and heading dependent on $\beta^{c}$.

Variances of position and rotation estimates are shown in Figure 5. As in the previous figure, the end of the measurement range is characterised by a sharp increase in the variance. Another very important finding is that the variance is not constant throughout the measurement range. The variance of rotation $m_{\beta }^{c}$ is characterised by an increase in the proximity of $0^{\circ }$ where the marker surface is parallel to the camera. The same effect can be observed in the variance of position $m_{x}^{c}$ although it is not that pronounced. This increase is caused by the pose ambiguity issue discussed in Section 1 and Section 2. The variance of $m_{z}^{c}$ is shown in logarithmic scale due to the large difference between the magnitude of the variance near the ends of the measurement range and the rest of the data.

**Figure 6:**
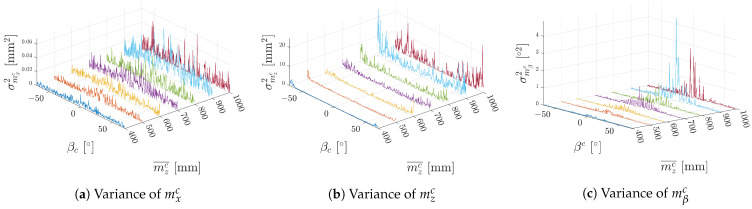
Variances in different distances from the camera dependent on yaw angle $\beta^{c}$. Measurements from different distances are colour coded.

To visualise the change in variances in dependence not only on the rotation of the marker but also on the distance from the marker, measurements from distances in $z^{c}$ ranging from 400 mm to 1000 mm and $x^{c}$ equal to zero are presented in Figure 6. To see details of the variances, the sharp changes at the edge of the measurement range are trimmed off in the following figure. The trend observed in Figure 5 is also visible in the measurements farther away from the marker only with an increase in magnitude. It can also be observed that the peak magnitude of the variance of $m_{\beta }^{c}$ is increasing at a higher rate than the other two values.

**Figure 7:**
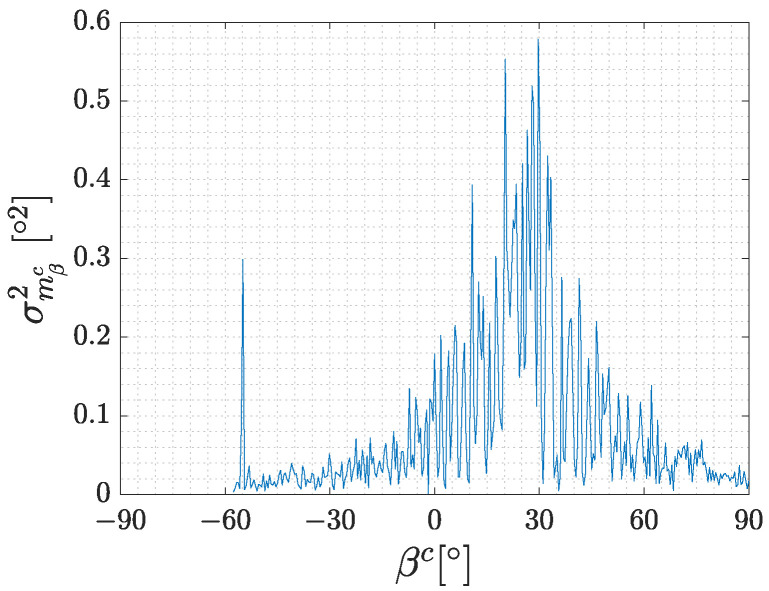
Peak of variance shift due to the marker offset.

The results of experiments in which the position of the marker in the camera coordinate system is non-zero in the direction of $x^{c}$ had trends in variance similar to the previous results. The most significant difference was the shift of the variance peak of $m_{\beta }^{c}$ as can be observed in Figure 7. This measurement was taken when the marker was moved 300 mm in the direction of $x^{c}$ and 600 mm in $z^{c}$. It can be seen that the peak of the variance is approximately at $30^{\circ }$ which corresponds to the situation where the normal vector of the marker points directly at the camera. This can be proven if we calculate the angle between the normal vector of the marker and the axis $z^{c}$, which is equal to $atan(m_{x}^{c}/m_{z}^{c})$ resulting in $29.5^{\circ }$ for the described situation.

### 4.3. Simulation Results

A large part of the development of modern robotic systems is done in simulations. Therefore, it is important to investigate whether the behaviour observed in real experiments is also present in the simulation environment. Gazebo robotic simulator [39] with the model of our robot equipped with the camera was used for the following experiments.

The camera sensor was set to the same resolution and field of view as the camera in the previous experiments. Radial and tangential distortion of the camera was neglected because it would be compensated in a calibration process anyway. In addition, Gaussian noise with a standard deviation of 0.01 was applied to the camera, so it would correspond to the noise present in the real camera. Due to the absence of shadows in the simulation, the marker was rotated only from $0^{\circ }$ to $90^{\circ }$ with an increment of one degree during each measurement.

**Figure 8:**
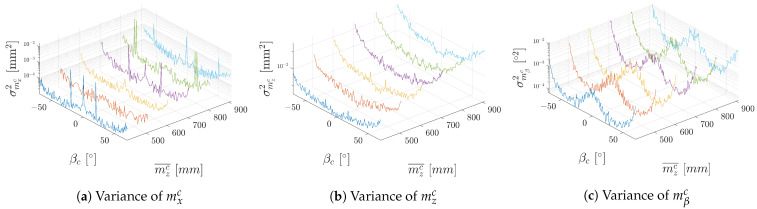
Variances in different distances from the camera dependent on yaw angle $\beta^{c}$ obtained from simulation in Gazebo. Measurements from different distances are colour coded.

The results of the experiments are presented in Figure 8b. In this set of experiments, the camera was moved from 400 mm to 1000 mm in direction of $z^{c}$ and positioned at 0 mm in $x^{c}$. To better visualise the trends in the variance, the *z* axes of the graphs are in logarithmic scale. We can observe the same trends in the results as in the real experiments, differing only in the magnitude of the variance. This difference is likely due to imperfections present in the real camera that are not reflected in the camera model in the simulation.

### 4.4. Conclusion of Measurements

The results of both simulation and real experiments support the previous findings presented in [5,30,31,32]. The effects of pose ambiguity, and precision degradation with increasing distance and at the ends of the measuring range are clearly visible in the data. Furthermore, the effect of the camera not being in line with the marker is described. The important finding is that the variance depends not only on the distance of the camera from the marker but also on the angle of the marker relative to the camera. Moreover, this effect is most visible in the variance of $\sigma_{m_{\beta }^{c}}$ where the variance between $40^{\circ }$ and $70^{\circ }$ is an order of magnitude lower than near the peak around $0^{\circ }$.

## 5. Analytical Models of Pose Variance

Based on the presented results, it was validated that the variance of the pose estimation obtained from the marker is not constant, but depends on the orientation and position of the marker relative to the camera. Furthermore, the differences in variance in a single experiment were significant enough that they cannot be neglected for proper state estimation using EKF.

The goal of this subsection is to derive models that capture the character of variance in individual axes that are relevant to our presented application. Presented models are analytical with minimum parameters, so they are easy to estimate and still represent the character of variance well enough. Models do not need to precisely fit the data for the correct behaviour of the EKF, more important is to correctly capture the course of variance and its order of magnitude.

### 5.1. Marker Area Normalisation

The variance of the camera pose relative to the marker depends on the yaw angle of the marker and its position, as shown in previous figures. However, if the variance model depended on the distance of the camera from the marker in a direction of $z^{c}$, this model would be usable only for markers of a specific size. The models can be independent of the size of the marker if they depend on the area of the marker in the image, since the larger marker farther away from the camera looks the same as the smaller marker closer to the camera, provided that they are both in focus. However, this also presents a problem since the area of the marker depends not only on its distance from the camera but also on its yaw angle relative to the camera. This can be solved by the area normalisation process presented in the next paragraph.

**Figure 9:**
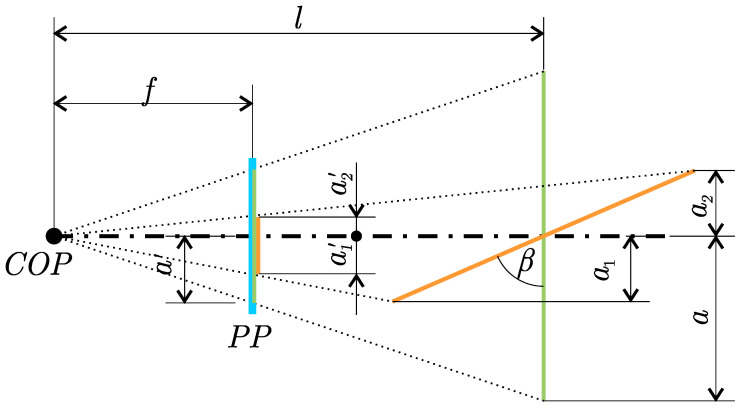
Top view of the rotated (orange) and equivalent (green) marker captured by the camera on the projection plane (blue).

**Figure 10:**
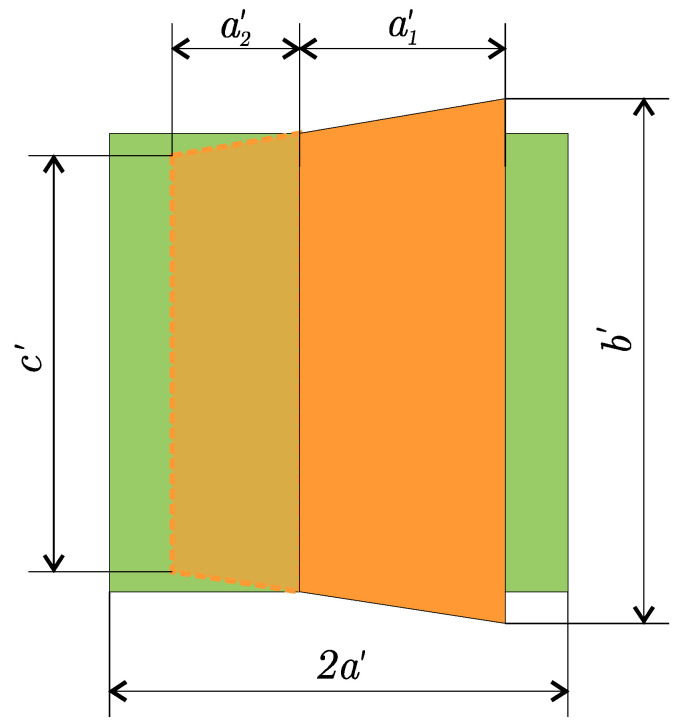
View of the rotated (orange) and equivalent (green) marker captured by the camera.

The goal of area normalisation of the marker is to convert the known image area $S_{\beta }$ of the marker that is rotated by a certain yaw angle β to the equivalent area $S_{n}$ of the marker that is in the same position and is not rotated. This situation in which the markers are captured by the camera is visualised from the top view in Figure 9, where the rotated marker with the edge size 2a is shown in orange and the equivalent non-rotated marker in green. These markers are projected on the projection plane PP of the camera that is at a focal distance *f* from the centre of the projection COP. The resulting image captured by the camera is shown in Figure 10.

The area of the non-rotated marker, representing the normalised area $S_{n}$, can be calculated as an area of a square with an edge size of $2a^{\prime }$, resulting in Equation (2), where *l* is the distance from the marker to the COP in the direction of the optical axis. The area of the rotated marker $S_{\beta }$ can be calculated as the area of the equilateral trapezoid with side lengths of $c^{\prime }$, $b^{\prime }$ and height of $a_{1}^{\prime }+a_{2}^{\prime }$. Side lengths $c^{\prime }$ and $b^{\prime }$ can be derived from Figure 9 as $c^{\prime }=\frac{2af}{l+a\sin\beta }$ and $d^{\prime }=\frac{2af}{l-a\sin\beta }$. The length of $a_{1}^{\prime }+a_{2}^{\prime }$ is obtained in a similar manner to $a_{1}^{\prime }=\frac{fa\cos\beta }{l-a\sin\beta }$ and $a_{2}^{\prime }=\frac{fa\cos\beta }{l+a\sin\beta }$. The resulting formula for the area of the rotated marker is represented by Equation (3).

(2) $$S_{n}=\frac{4a^{2}f^{2}}{l^{2}}$$

(3) $$S_{\beta }=\frac{4a^{2}f^{2}l^{2}\cos\beta }{{\left(l^{2}-a^{2}\sin^{2}\beta \right)}^{2}}$$

Finally, from the ratio of the area of the non-rotated and rotated markers, we can obtain Formula (4) for the conversion of the measured rotated marker area to the normalised marker area.

(4) $$\frac{S_{n}}{S_{\beta }}=\frac{{\left(l^{2}-a^{2}\sin^{2}\beta \right)}^{2}}{l^{4}\cos\beta }\Rightarrow S_{n}=\frac{{\left(l^{2}-a^{2}\sin^{2}\beta \right)}^{2}}{l^{4}\cos\beta }S_{\beta }$$

In case the marker is not positioned on the optical axis, Equation (4) is modified to Equation (5), where *x* represents the offset of the marker from the optical axis.

(5) $$S_{n}=\frac{{\left(l^{2}-a^{2}\sin^{2}\beta \right)}^{2}}{l^{3}\left(l\cos\beta +x\sin\beta \right)}S_{\beta }$$

**Figure 11:**
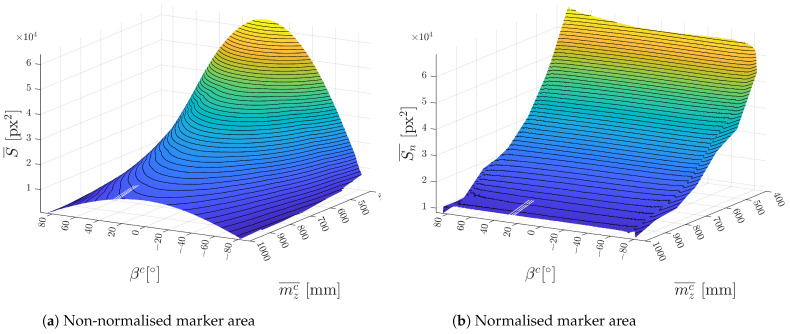
Dependency of a marker area on angle $\beta^{c}$ and distance $\bar{m_{z}^{c}}$.

The result of area normalisation is shown in Figure 11 where the average measured $\bar{S}$ and normalised $\bar{S_{n}}$ areas for each individual set of 100 photos are compared side by side. It can be seen that the area in Figure 11b is dependent only on the distance and not the angle $\beta^{c}$.

### 5.2. Model of $m_{\beta }^{c}$ Variance

The analytical model that describes the course of variance of $m_{\beta }^{c}$ should be designed so that it can adequately capture the character of the variance and be simple enough, with minimum parameters, that it can be easily estimated on measured data. The following significant characteristics that the model should consider can be deduced from Figure 5c and Figure 6c:

- The variance increases sharply at the edge of the detectability of the marker.
- The variance has a Gaussian shape around the point where the normal vector of the marker points towards the camera.
- The peak of the variance increases with increasing distance from the marker and consequently with a decreasing area of the detected marker.

Based on the mentioned criteria, an analytical function in the form of

$\sigma_{m_{\beta }^{c}}^{2}=f\left(m_{\beta }^{c},S_{n},m_{x}^{c},m_{z}^{c}\right)$ can be proposed. The resulting function (6) consists of the power function part describing the growth of the variance with decreasing marker area, the Gaussian part capturing the peak of variance, the part capturing the increase in variance at the edge of detectability and an offset. The angle ϕ expressed in Equation (7) is the offset of $m_{\beta }^{c}$ angle by the angle that represents the mutual position of the camera and the marker as described in Section 4.2. The model has five parameters $p_{1}$ to $p_{5}$ that must be estimated.

(6) $$\sigma_{m_{\beta }^{c}}^{2}=p_{1}S_{n}^{-p_{2}}e^{-\frac{\phi }{p_{3}}}+\left|\frac{p_{4}}{90-|\phi |}\right|+p_{5}$$

(7) $$\phi =m_{\beta }^{c}-atan\left(m_{x}^{c}/m_{z}^{c}\right)$$

**Figure 12:**
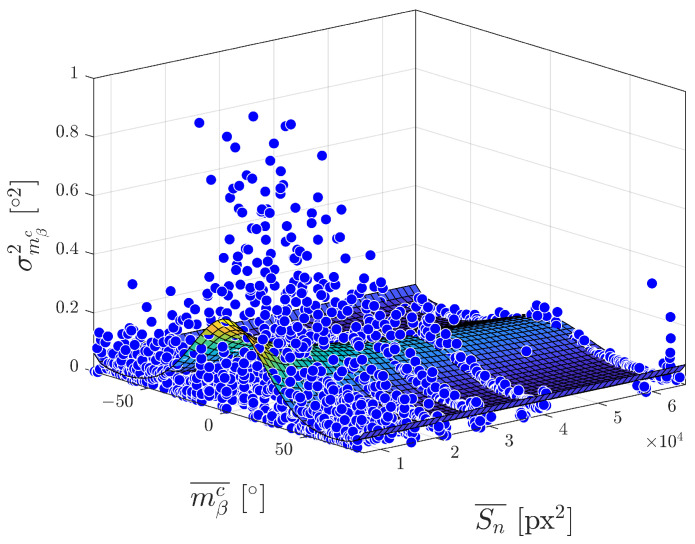
Model of $\sigma_{m_{\beta }^{c}}^{2}$ estimated on real data.

Figure 12 shows an analytical model fitted to data from the real experiment presented in Section 4.2. Parameters were estimated using the Levenberg–Marquardt algorithm for solving the nonlinear least squares problem with the goodness of fit expressed by RMSE equalled to $0.1000^{\circ^{2}}$.

### 5.3. Models of $m_{x}^{c}$ and $m_{z}^{c}$ Variances

The following significant characteristics can be deduced based on Figure 5a,b and Figure 6a,b.

- Variance sharply increases at the edge of the detectability of the marker.
- The variance increases with increasing distance from the marker and consequently with decreasing detected marker area.
- The variance is not significantly dependent on the yaw angle $m_{\beta }^{c}$.

The following analytical model given by (8) is based on previously mentioned characteristics of $m_{x}^{c}$ and $m_{z}^{c}$ variances. Since both variances show similar characteristics, only a single model will be used for both of them. The model consists of a part describing the growth of variance based on the decreasing area of the marker captured in the image. The denominator captures the increase in variance near the end of the measuring range and the last part is the offset of the model. The angle ϕ is given by Equation (7). The model has three parameters $p_{1}$ to $p_{3}$.

(8) $$\sigma_{m_{x/z}^{c}}^{2}=\frac{p_{1}S_{n}^{-p_{2}}}{90-|\phi |}+p_{3}$$

**Figure 13:**
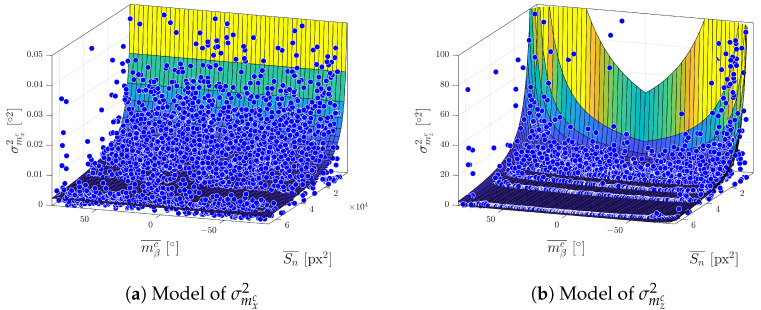
Models of $\sigma_{m_{x}^{c}}^{2}$ and $\sigma_{m_{z}^{c}}^{2}$ estimated on real data.

Figure 13 shows the analytical models fitted to the data from the real experiment presented in Section 4.2. The parameters were estimated using the same method as in the previous Section 5.2 with the goodness of fit expressed by RMSE equalled to $0.0076^{\circ^{2}}$ for model of $\sigma_{m_{x}^{c}}^{2}$ and $1.3596^{\circ^{2}}$ for model of $\sigma_{m_{z}^{c}}^{2}$.

## 6. Practical Application of Variance Models in 2D Mobile Robot Localisation

**Figure 14:**
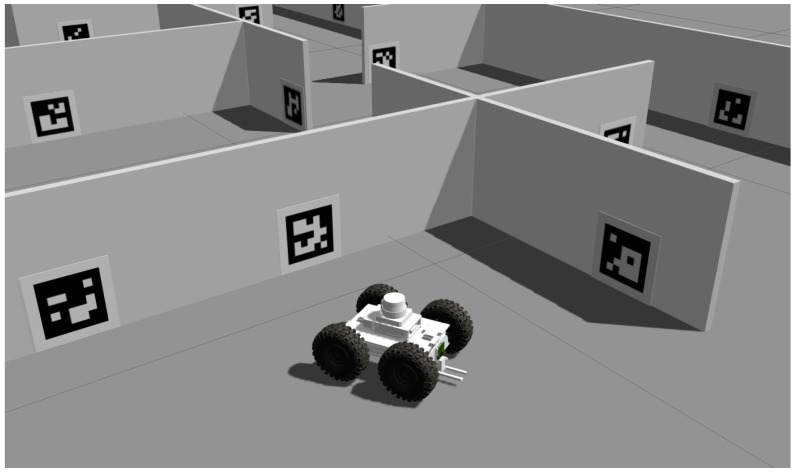
Simulation environment with a robot created in Gazebo.

The following section presents an approach describing one of the ways of using the presented variance models in the mobile robot localisation problem. As described in Section 1, our research focused on the localisation of a four-wheel skid-steered mobile robot in a known environment with planar fiducial markers placed at known positions. The mobile robot in question is equipped only with a colour camera with a resolution of 1280 × 720 px, IMU and wheel encoders. The described use case is implemented in the Gazebo simulator in order to have full control over the experiment and its parameters and to have a ground truth reference for comparison. The robot in the simulation environment is presented in Figure 14.

### 6.1. Parameter Estimation on User Data

In Section 5, the parameters of the observation noise models were estimated on a large dataset of measurements with a precisely set yaw angle of the marker that was considered ground truth. This approach is not very practical in a real scenario due to its time consumption and the requirements for the preparation of the experiment. Therefore, with knowledge of significant features of variance models, only a subset of measured data is needed to estimate the required parameters in all models.

Because the measured variance is symmetric around $0^{\circ }$, measurements can be made in only one-half of the measurement range and then mirrored. For the model of $m_{\beta }^{c}$ the measurements are needed at the yaw angle of $0^{\circ }$ to capture the increase in variance at this angle of rotation. The next significant data points are around the angle of $15^{\circ }$ to capture the spread of the peak. Other points are around $40^{\circ }$ capturing the value of minimal variance and the final points are near the edge of detectability around $70^{\circ }$ to capture the increase of variance in this area. The data points mentioned above will also serve well enough for the estimation of the parameters of the variance models of $m_{x}^{c}$ and $m_{z}^{c}$. All data points at different yaw angles should be measured in at least five points throughout the measurement range in the direction of the $z^{c}$ axis. It is recommended to take at least 100 photos of the marker in each configuration.

### 6.2. Transformation of Measurements to Global Coordinate System

Each estimated pose from a marker can be considered as a measurement with a multivariate Gaussian distribution defined as $N(\mu_{m^{c}},\Sigma_{m^{c}})$, where $\mu_{m^{c}}$ is a vector of mean values representing measured positions and rotation, and $\Sigma_{m^{c}}$ is a covariance matrix with modelled variances on the main diagonal.

To transform a multivariate Gaussian distribution, a first-order linear transformation can be used as described in [40] by Equations (9) and (10).

(9) $$c^{g}=f\left(m^{c},m^{g}\right)$$

(10) $$\Sigma_{y}=\frac{\partial f(x)}{\partial x}\Sigma \frac{\partial f{\left(x\right)}^{T}}{\partial x}$$

The function $f(m^{c},m^{g})$ represents a transformation of the position and orientation of the camera and consequently of the robot itself from the camera coordinate system to the global coordinate system. The known pose of the marker in the global coordinate system $m^{g}$ and in the camera coordinate system $m^{c}$ needs to be combined using the geometric transformations in Equations (11)–(13) to obtain the pose of the camera in the global coordinate system $c^{g}$.

(11) $$c_{x}^{g}=m_{x}^{c}\cos\left(m_{\beta }^{g}+m_{\beta }^{c}\right)-m_{z}^{c}\sin\left(m_{\beta }^{g}+m_{\beta }^{c}\right)+m_{x}^{g}$$

(12) $$c_{z}^{g}=m_{x}^{c}\sin\left(m_{\beta }^{g}+m_{\beta }^{c}\right)+m_{z}^{c}\cos\left(m_{\beta }^{g}+m_{\beta }^{c}\right)+m_{z}^{g}$$

(13) $$c_{\beta }^{g}=m_{\beta }^{g}+m_{\beta }^{c}$$

The term $\frac{\partial f(x)}{\partial x}$ in (10) represents the Jacobian of $f(m^{c},m^{g})$. Considering that normal distributions of the measurements represented by $m^{c}$ and $m^{g}$ are independent, Equation (10) can be rewritten in the following form:

(14) $$\Sigma_{c^{g}}=\frac{\partial f(m^{c},m^{g})}{\partial m^{c}}\Sigma_{m^{c}}\frac{\partial f{\left(m^{c},m^{g}\right)}^{T}}{\partial m^{c}}+\frac{\partial f(m^{c},m^{g})}{\partial m^{g}}\Sigma_{m^{g}}\frac{\partial f{\left(m^{c},m^{g}\right)}^{T}}{\partial m^{g}}$$

Moreover, Jacobians $\frac{\partial f(m^{c},m^{g})}{\partial m^{c}}$ and $\frac{\partial f(m^{c},m^{g})}{\partial m^{g}}$ are represented by the following matrices:

(15) $$\frac{\partial f(m^{c},m^{g})}{\partial m^{c}}=\left[\begin{array}{ccc} \cos(m_{\beta }^{g}+m_{\beta }^{c}) & -\sin(m_{\beta }^{g}+m_{\beta }^{c}) & -m_{x}^{c}\sin\left(m_{\beta }^{g}+m_{\beta }^{c}\right)-m_{z}^{c}\cos\left(m_{\beta }^{g}+m_{\beta }^{c}\right)\\ \sin\left(m_{\beta }^{g}+m_{\beta }^{c}\right) & \cos\left(m_{\beta }^{g}+m_{\beta }^{c}\right)m_{x}^{c} & \cos\left(m_{\beta }^{g}+m_{\beta }^{c}\right)-m_{z}^{c}\sin\left(m_{\beta }^{g}+m_{\beta }^{c}\right)\\ 0 & 0 & 1 \end{array}\right]$$

(16) $$\frac{\partial f(m^{c},m^{g})}{\partial m^{g}}=\left[\begin{array}{ccc} 1 & 0 & -m_{x}^{c}\sin\left(m_{\beta }^{g}+m_{\beta }^{c}\right)-m_{z}^{c}\cos\left(m_{\beta }^{g}+m_{\beta }^{c}\right)\\ 0 & 1 & m_{x}^{c}\cos\left(m_{\beta }^{g}+m_{\beta }^{c}\right)-m_{z}^{c}\sin\left(m_{\beta }^{g}+m_{\beta }^{c}\right)\\ 0 & 0 & 1 \end{array}\right]$$

### 6.3. Fusion of Pose from Multiple Markers

If multiple markers are detected in the image, their multivariate Gaussian distributions can be combined to improve the position estimate using their product [41] in the form of Equations (17) and (18).

(17) $$\mu ={\left(\sum_{n=1}^{N}\Sigma_{n}^{-1}\right)}^{-1}\left(\sum_{n=1}^{N}\Sigma_{n}^{-1}\mu_{n}\right)$$

(18) $$\Sigma ={\left(\sum_{n=1}^{N}\Sigma_{n}^{-1}\right)}^{-1}$$

This fused estimate with its covariance can be used as one of the inputs in the EKF or other state estimators in the form of the observation noise matrix $R_{k}$.

### 6.4. EKF Localisation

**Figure 15:**
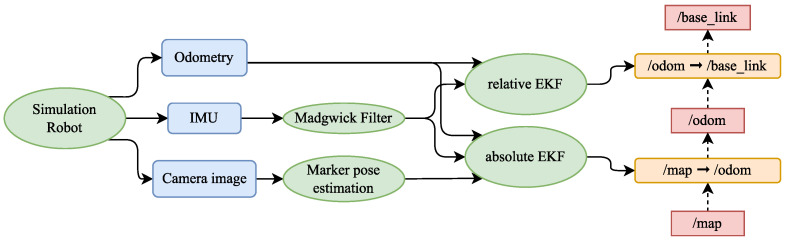
Diagram of localisation pipeline with transformations.

As a final step, an EKF is used to fuse all sensory data and provide an estimate of the absolute position of the mobile robot in the environment. The experiment was carried out using the ROS 1 framework [42] in the Gazebo simulation environment. The simplified diagram that describes the entire localisation pipeline is shown in Figure 15.

The outputs from the simulation were in the form of odometry calculated using a differential drive model, data from the simulated IMU that were processed using the Madgwick filter [43] and camera images that were processed using the method described in this article. These sensor data were then sent as input to the EKF.

According to ROS conventions [44] three coordinate systems (frames) were used, map, odom and base_link. The transformation from odom to base_link is provided by the relative EKF that fuses data from the IMU and odometry. Absolute EKF is used to provide the transformation from map to odom based on data from the IMU, odometry and the global position estimate from fiducial markers. EKFs were implemented using a node from the *robot_localization* package [45].

### 6.5. Results

**Figure 16:**
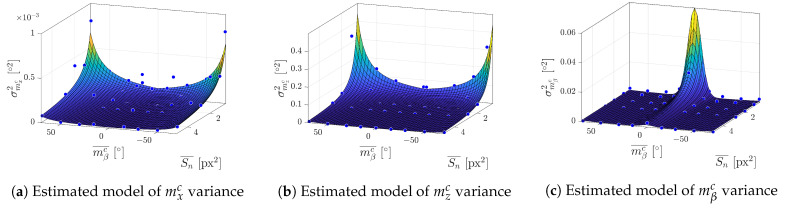
Parameter estimation on user data.

Figure 16 presents the variance models estimated from the simulation data. Data points were selected according to the recommendations in Section 6.1 from five different distances 400, 500, 600, 800 and 1100 mm, and five yaw angles of the marker $0^{\circ },15^{\circ },30^{\circ },50^{\circ }$ and $70^{\circ }$. Parameters were estimated using the Levenberg–Marquardt algorithm for solving the nonlinear least squares problem.

**Figure 17:**
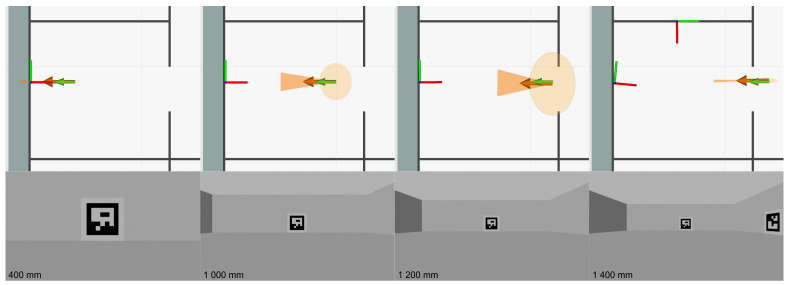
Visualisation of covariance error ellipse based on distance and number of visible markers (the orange arrow represents pose estimated from markers with its covariance, the green arrow represents the ground truth position of the mobile robot and marker positions are represented by coordinate axes).

**Figure 18:**
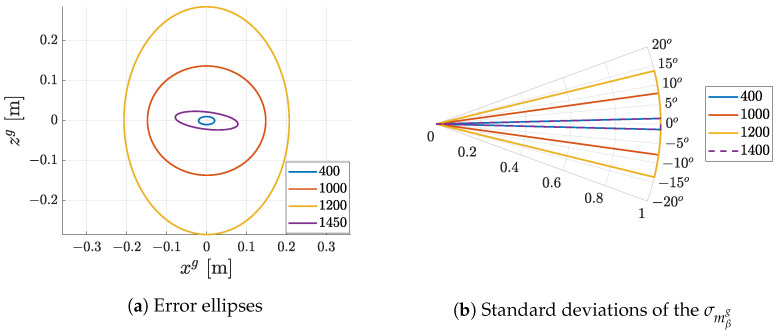
Comparison of standard deviations based on different distances $m_{z}^{c}$ from the marker.

To visualise the effect of presented variance models, the robot with the camera was placed 400, 600, 1000 and 1200 mm from the marker, and the resulting observation noise in the form of covariance matrices at each position is visualised as error ellipses in Figure 17 and compared in Figure 18. The covariance matrix of the marker positions in the global coordinate system was set to zero; therefore, only the influence of variance models is visible. It can be observed that, with increasing distance from the marker, the error ellipse increases up to the point where two markers are visible. When multiple markers are visible, as in Figure 17 for a robot position of 1400 mm, their pose estimates can be fused. This results in a more accurate pose estimate in which the resulting position of the robot is closer to the ground truth.

**Figure 19:**
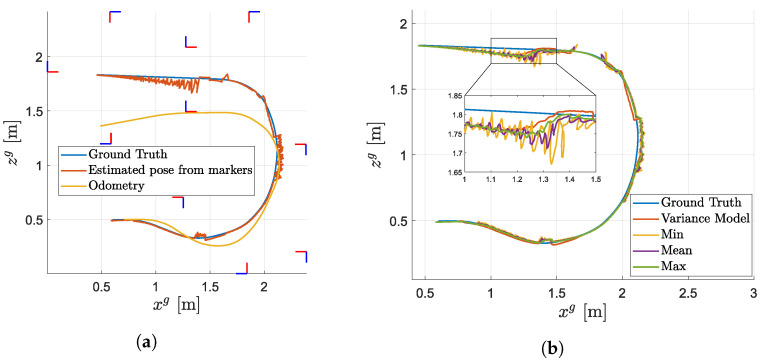
Results from EKF localisation. (**a**) Comparison of odometry and position estimated from markers (shown as marker axes) with ground truth position and depiction of visible markers. (**b**) Comparison of position estimated by EKF using different variance models for the estimated pose from markers.

**Figure 20:**
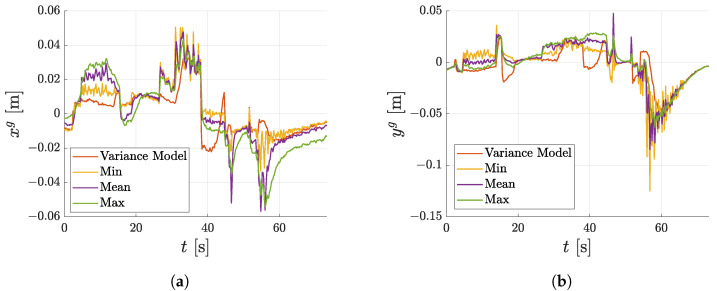
Comparison of differences from the ground truth in direction of $x^{g}$ and $y^{g}$ axes. (**a**) Difference from ground truth in direction of $x^{g}$. (**b**) Difference from ground truth in direction of $y^{g}$.

In the last experiment, the mobile robot was driven manually in the simulation environment and its absolute position in the global coordinate system was estimated using the localisation pipeline described in Section 6.4. The experiment was carried out four times on the same data set, differing only in the variance model used. In the first experiment, the model of variance, as described in this paper, was used. In the following experiment, the variances of the pose estimate were set to a fixed value corresponding to the minimal, mean and maximal values of the measured variances used to estimate the individual variance models shown in Figure 16. The results of this experiment are shown in Figure 19 where the inputs to the EKF, odometry and estimated pose from the markers are compared with the ground truth in Figure 19a and the individual estimated poses resulting from the different settings of the variance models are compared in Figure 19b. The ground truth position was obtained directly from the simulation in Gazebo. Figure 20 presents a better visualisation of the differences among individual experiments as errors from the ground truth position for the $x^{g}$ and $z^{g}$ axes. The RMSE values, evaluated between the ground truth position and position obtained while using different variance models, are summarised in Table 1 for $x^{g}$ and $y^{g}$ axes.

## 7. Discussion

The methods presented in the previous sections proposed a straightforward way of estimating observation noise for pose estimates obtained from planar markers using analytical models. This pose estimate with its covariance matrix can consequently be used, for example, for localisation using the EKF as was presented.

The benefit of the proposed approach is that it eliminates the need for manual setting of the observation noise which can be estimated using the simple method with a relatively low number of measurements described in Section 6.1. The accuracy of the estimation depends on the position of the selected data points and their amount. However, with an increasing number of data points, the whole process of acquiring these data points becomes more time consuming.

The next key outcome is visible in Figure 19b and Table 1 where the position from localisation using the proposed variance models outperforms the estimates in which the fixed variance was used for the estimate of the marker pose. This is because the presented analytical models capture the true nature of the variances of the marker. Therefore, the resulting pose is less susceptible to less accurate pose estimates received from markers compared to situations where the variances are set at a constant value. This is more apparent in situations where the robot is farther away from the marker or the marker is less detectable, as shown in the zoomed section in Figure 19b where the proposed method is less susceptible to the noisy pose estimate from the marker compared to other methods. Setting the constant variances to either the minimal or maximal value causes the state estimator to trust the measurement less when it is accurate or more reliable when it is not.

While the approach we presented was demonstrated for 2D localisation, it can be generalised and applied to an arbitrary situation in a 3D environment. Moreover, the step of fusing the pose estimates from multiple markers can be integrated into the Kalman filter itself, rather than being a separate process. However, in this scenario, the Kalman filter must be able to handle a varying number of inputs, depending on the number of visible markers.

Future research should focus on methods for the fusion of estimates from multiple markers. Although the proposed method resulted in overall better performance compared to settings with the fixed variance of pose estimate, the compared methods provided better estimates in certain sections. This is likely caused by a violation of our assumption that individual pose estimates from multiple markers in the image are Gaussian and independent. Another consequence of this assumption is that, in cases where many markers are visible, the resulting product of Gaussian distributions would have unrealistically low variances.

An area for further research would be to enhance the proposed variance models, which are currently estimated for static camera situations. However, other factors such as motion blur resulting from the robot’s movement and camera vibrations can degrade the captured image and, as a result, the estimated pose from the fiducial marker. These influences must be considered in order to improve the accuracy of the pose estimates and the overall performance of the localisation system.

## 8. Conclusions

This paper presents a method for estimating the variance of the pose estimate from planar fiducial markers. The estimated variance is based on analytical models that correctly capture the varying nature of the variance that was experimentally validated. These models are simple enough to be easily estimated on a relatively small amount of data and still capture the variable nature of the pose estimate variances. The method used here can be applied to the problem of mobile robot localisation as was presented in Section 6.4. Here, our method of setting the observation noise of EKF based on variance models proved to be superior to experiments with variances set to a constant value.

## Figures and Tables

**Table 1:** Comparison of RMSE for individual variance setting in direction of $x^{g}$ and $y^{g}$ axes.

	$x^{g}$ RMSE [m]	$z^{g}$ RMSE [m]
Variance Model	0.0128	0.0161
Min	0.0154	0.0215
Mean	0.0192	0.0207
Max	0.0223	0.0203

## Data Availability

Data is unavailable due to privacy restrictions.
